# The Tol-Pal System Promotes Pseudoiodinine Production by Enhancing Bacterial Growth in *Pseudomonas mosselii*

**DOI:** 10.3390/microorganisms14092079

**Published:** 2026-09-17

**Authors:** Ruihuan Yang, Xiangning Du, Heng Zhang, Jiacan Yang, Zhuoyi Huang, Ning Xu, Lifang Zou, Gongyou Chen

**Affiliations:** 1 National Key Laboratory of Cotton Bio-Breeding and Integrated Utilization, Henan Joint International Laboratory for Crop Multi-Omics Research, School of Life Sciences, Henan University, Kaifeng 475000, China; 2 Shanghai Collaborative Innovation Center of Agri-Seeds/State Key Laboratory of Microbial Metabolism, School of Agriculture and Biology, Shanghai Jiao Tong University, Shanghai 200240, China; 3 State Key Laboratory of Agricultural and Forestry Biosecurity, Ministry of Agriculture and Rural Affairs of the People’s Republic of China Key Laboratory of Pest Monitoring and Green Management, College of Plant Protection, China Agricultural University, Beijing 100193, China

**Keywords:** pseudoiodinine, *Pseudomonas mosselii*, tol-pal system, growth, production

## Abstract

Pseudoiodinine is an antimicrobial compound produced by *Pseudomonas mosselii* that exhibits strong inhibitory activity against bacterial and fungal pathogens of rice, highlighting its potential as a green biopesticide. However, the regulatory mechanisms underlying pseudoiodinine production remain poorly understood. In this study, genome-wide random mutagenesis identified two insertion mutants of 277-3 and 277-23 that completely abolished antagonistic activity against *Xanthomonas oryzae* pv. *oryzicola* (*Xoc*) RS105, with transposon insertions mapped to *tolB* and *tolR*, respectively. Further analysis showed that mutations in *tolB* and *tolR* significantly impaired bacterial growth during the exponential phase, suggesting that the Tol-Pal system is required for the optimal growth of *P. mosselii*. Consistently, overexpression of the *tol-pal* operon resulted in a 3.2-fold increase in pseudoiodinine production compared with the original strain. These findings indicate that the Tol-Pal system contributes to pseudoiodinine production primarily by maintaining efficient bacterial growth. Collectively, this study provides the first insight into the Tol-Pal system in promoting pseudoiodinine production and suggests its potential application in engineering *P. mosselii* strains.

## 1. Introduction

Plants constantly face biotic stress from a variety of pathogens, including bacteria, fungi, and oomycetes in their living environments, which severely threatens global food security and sustainable agriculture [1,2]. Rice (*Oryza sativa* L.), one of the most important staple crops worldwide [3], was hampered by multiple devastating diseases, including bacterial leaf blight (BLB), bacterial leaf streak (BLS), and rice blast, which were caused by *Xanthomonas oryzae* pv. *oryzae* (*Xoo*), *X. oryzae* pv. *oryzicola* (*Xoc*), and *Magnaporthe oryzae*, respectively [4]. Although breeding resistant cultivars represented an effective strategy for controlling rice diseases [5], the lack of major resistance genes made it necessary to continue to use chemical pesticides, which has resulted in pathogen resistance, environmental pollution, and pathogen resurgence [6]. Therefore, environmentally friendly and sustainable disease management strategies are urgently needed. Biological control as an environmentally friendly approach has gained considerable importance. At present, various biocontrol agents (BCAs) derived from *Bacillus*, *Pseudomonas*, *Burkholderia*, *Streptomyces*, and *Trichoderma* have been explored for plant disease suppression through diverse mechanisms [7]. Among these mechanisms, the production of antimicrobial metabolites has represented one of the most important strategies employed by beneficial microorganisms. Several microbial-derived antimicrobial compounds have been developed for plant disease control, highlighting their potential as green biopesticides [8].

Pseudomonas species are ubiquitous in diverse environments, including soil, water, and plant-associated niches, and are notable for producing a wide array of antimicrobial metabolites (AMs) [9]. These metabolites, including 2,4-diacetylphloroglucinol (2,4-DAPG), phenazine, Pyrrolnitrin (Prn), Pyoluteorin (Plt), cyclic lipopeptides (CLPs), pseudomonic acid, and hydrogen cyanide (HCN), exhibit potent inhibitory activities against various plant pathogens and contribute to global agricultural sustainability [8]. However, despite extensive efforts in discovering microbial-derived antimicrobial compounds, the availability and field application efficiency of commercial green biopesticides remain limited. Among these beneficial pseudomonads, *Pseudomonas mosselii*, a member of the *P. putida* group [10], has attracted increasing attention due to its plant-associated lifestyle and metabolic versatility. This species has shown remarkable potential in biocontrol against plant diseases and promoting plant growth. *P. mosselii* has been reported to strongly inhibit tumor formation in tomato stems by inhibiting the growth of *Agrobacterium tumefaciens* [11]. In addition, reports also displayed that *P. mosselii* exhibited significant inhibitory activity against *Magnaporthe oryzae* by producing xantholysin analogs [12]. Moreover, *P. mosselii* ACO-140 exhibited excellent phosphate solubilizing and nitrogenase activity, and it can synthesize indole-3-acetic acid, which promotes plant growth [13].

In our previous work, we isolated *P. mosselii* strain 923 from rice rhizosphere soils of paddy fields; the antimicrobial compound was identified as pseudoiodinine, which was effective in controlling rice blast, BLB, and BLS [14]. Notably, pseudoiodinine showed significantly lower MIC and EC_50_ values than phenazine-1-carboxylic acid (PCA), the active component of Shenqinmycin, a commercially registered biopesticide in China [14,15]. These findings highlight the potential of pseudoiodinine as a promising environmentally friendly biopesticide. Further studies revealed that the GacA-RsmY/Z-CsrA1/2/3 regulatory cascade played an important role in modulating pseudoiodinine biosynthesis in *P. mosselii* [14]. Increasing evidence indicated that secondary metabolism was not solely determined by transcriptional regulatory circuits, such as the Gac/Rsm pathway [16], quorum sensing [17], and specific transcriptional regulators, but was also closely associated with bacterial physiological status, including growth rate, nutrient availability, cellular energy availability, environmental stresses, and envelope homeostasis [18,19]. However, whether bacterial envelope-associated systems involved in maintaining cellular fitness participate in pseudoiodinine production remains largely unknown.

The Tol-Pal system is a highly conserved envelope-associated system in most Gram-negative bacteria that plays essential roles in maintaining bacterial growth and cell envelope integrity [20]. This multi-protein complex is composed of TolQ, TolR, TolA, TolB, and Pal envelope proteins [21], traversing the outer membrane, periplasmic space, and inner membrane [22]. TolA as a crucial inner membrane protein and has the capacity to form a TolQ-TolR-TolA complex with TolR and TolQ [23]. The TolB protein can interact with the Pal protein localized in the outer membrane [24]. Additionally, novel findings revealed that the TolQ-TolR-TolA integral inner membrane protein complex can modulate the interaction between TolB and Pal proteins near the outer membrane [25], continually influencing the levels of Pal binding to the cell wall, particularly at the cell septum [26]. The Tol-Pal system was originally characterized in *Escherichia coli* [27] and subsequently was reported in many other bacterial pathogens, including *Pseudomonas putida* [28], *P. aeruginosa* [29], *Vibrio cholerae* [30], *Salmonella Choleraesuis* [31], *S. Typhimurium* [32], and *Haemophilus ducreyi* [33]. In particular, the *tol-pal* genes in *Erwinia chrysanthemi* have been reported to sustain the virulence and activity of pectinolytic enzymes in the plant cell wall [34]. There is increasing evidence that the Tol-Pal system plays numerous biologic functions in various bacterial species, which includes cell morphology and virulence [34], motility and cell growth [22], biofilm formation and antibiotic resistance [21], outer membrane integrity, and outer membrane vesicle biogenesis [31]. The latest research has demonstrated that the Tol-Pal system connects envelope layers to maintain envelope integrity [35]. Subsequently, researchers found that the Tol-Pal complex plays a key role in maintaining outer membrane lipid homeostasis throughout the cell [36]. Strains lacking any *tol*-*pal* genes of *tolA*, *tolB*, *tolQ*, *tolR*, and *pal* have been known to exhibit diversified phenotypes. Previous studies have showed that a mutant *tolA* gene in *E. coli* exhibited attenuated biofilm formation ability, virulence, and resistance to environmental stresses [21]. Similarly, the lack of *tolA*, *tolB*, and *tolR* genes mainly inhibited cell growth and motility, impaired envelope integrity, and reduced virulence in *S. Choleraesuis* [31]. Taken together, the Tol-Pal system plays crucial roles in bacterial cell division and the stability of the outer membrane, determining its functions in bacterial survival and pathogenesis. However, the function of the Tol-Pal system in *P*. *mosselii* remains poorly understood.

In this study, with the aim of exploring the roles of *tol-pal* genes in the pseudoiodinine production of *P. mosselii*, we obtained two insertion mutants in the *tolB* and *tolR* genes via genome-wide transposon mutagenesis. We found that these two genes were involved in the cell growth of *P. mosselii*. Importantly, we engineered an overexpressing strain of Δ*csrA1A2A3*-pBS-*tol-pal* that can increase pseudoiodinine production by 3.2-fold. In general, our findings demonstrated that TolB and TolR were indispensable for *P. mosselii* cell growth and that overexpressing the *tol*-*pal* operon could improve pseudoiodinine production. Elucidating the roles of the Tol-Pal system in pseudoiodinine production will help us to further engineer *P. mosselii*, which will facilitate its application in agriculture and natural product pharmaceuticals.

## 2. Materials and Methods

### 2.1. Plasmids, Strains, and Growth Conditions

Plasmid pRL1063a was kindly offered by Dr. Gang Wang (School of Life Sciences, Henan University). Plasmid pBSPPc was provided by Dr. Shuangjun Lin (School of Life Sciences and Biotechnology, Shang Hai Jiao Tong University). The genetically modified strain GMS of Δ*csrA1A2A3*-pBSPPc-P2064-RBS-Pseu-ORF and the mutant strain Δ*csrA1A2A3* were obtained from our previous research, respectively [14]. Phytopathogenic *Xanthomonas* spp. including *X. oryzae* pv. *oryzae* (*Xoo*) PXO99^A^ and *X. oryzae* pv. *oryzicola* (*Xoc*) RS105 were cultivated in nutrient agar (NA) at 28 °C. The GMS strain and its mutants were cultured in tryptic soy broth (TSB; 30 g/L) medium at 30 °C. *Escherichia coli* DH5α was grown in Luria-Bertani (LB) medium at 37 °C. When required, antibiotics were added to the media at the following final concentrations: gentamicin (Gm, 20 μg/mL), kanamycin (Km, 25 μg/mL), and carbenicillin (Carb, 100 µg/mL). The plasmids and strains used in this study are presented in Table S1.

### 2.2. Tn5 Mutagenesis Screening and Identification

Mutagenesis of the GMS strain with Tn5-1063 was conducted as follows: the suicide plasmid pRL1063a carrying Tn5-1063 was introduced into GMS by parental mating. Briefly, strains pRL1063a and GMS were cultured in LB to an OD_600_ of 2.0 and 1.0, respectively. Their cell suspensions were prepared by centrifugation at 3500 rpm for 5 min, washing twice with sterile distilled water, and resuspended with 1 mL sterile distilled water. They were mixed in a 1:10 ratio, pellets were harvested by centrifugation at 3500 rpm for 5 min, and they were suspended in 100 μL sterile distilled water. Then, they were co-cultured at 28 °C for 12 h to facilitate cell fusion. The bacterial lawn was washed with 3 mL sterile distilled water, and each 100 μL of the cells was set on plates containing Km and Gm and incubated at 28 °C for 2 days. The colonies were moved individually to the NA medium containing *Xoc* RS105 and Km + Gm to screen for the mutants that appeared to strengthen or weaken antagonistic activity against *Xoc* RS105.

The rescued cloning of the Tn5-1063 transposon insertion site in the GMS genomic DNA was given according to the published protocols with modifications [37]. In brief, the genomic DNA from the chosen mutants was digested by *Eco*RI and then were self-ligated with T4 DNA ligase (5 units; Thermo Fisher Scientific, Waltham, MA, USA) at 22 °C for 12 h. Then they were introduced into *E. coli* DH5α competent cells and cultured on LB containing Km and Gm. Colonies containing Tn5 were confirmed by PCR with Tn5-F and Tn5-R, and the insertion sites were confirmed by sequencing using the forward or reverse Tn5-1063 transposon-specific primers in Table S2.

### 2.3. Generation of tolB and tolR Complementation Strains

Here we selected two mutants of 277-3 and 277-23 to complement. Briefly, *tolB*, *tolR*, and the *tolQ* promoter were amplified and cloned in pBSPPc with the ClonExpress II One Step Cloning Kit (Vazyme, Nanjing, China) to obtain plasmids pBS-*tolB* and pBS-*tolR*, respectively (Table S1). The primers used to amplify and identify sequences of each mutant are listed in Table S2. Constructs then were introduced into the corresponding mutants by electroporation and tested for bacteriostasis.

### 2.4. Quantitative Analysis of Pseudoiodinine Production

Pseudoiodinine was extracted and quantified according to Yang et al. (2023) [14] with modifications. Briefly, before pseudoiodinine extraction, the fermentation cultures were adjusted to the same OD_600_ to normalize bacterial biomass among the GMS, mutant, and overexpression strains. Equal volumes of the OD_600_-normalized cultures were subsequently subjected to pseudoiodinine extraction and quantification. Ultra performance liquid chromatography (UPLC) analyses were performed by a Thermofisher Vanquish Flex UHPLC system (Thermo Fisher) with an C18 column (150 × 4.6 mm, 5 μm, Agilent Technologies, Santa Clara, CA, USA). The detector was set to 500 nm, and the column temperature was kept at 30 °C. Linear gradient elution with a flow rate of 0.4 mL/min using solvents A (water) and B (methanol) was conducted as follows: 5% B for 4 min, 5% B to 20% B for 21 min, changing to 100% B for 1 min, holding 100% B for 6 min, and finally returning to the initial conditions (5% B) for 1 min, then holding 5% B for 3 min, with a total run time of 36 min. A standard curve with purified pseudoiodinine concentrations dissolved in water was used for quantification according to the following formula: A (peak area) = 9.4803 pseudoiodinine (mM) + 2.2671 (R^2^ = 0.9924).

### 2.5. Bacterial Growth Curve Assays

The GMS and its insertion mutants (277-3 and 277-23) and other strains were cultivated in LB medium overnight, respectively. Their OD_600_ were adjusted to 0.1 and dispensed into a 100-well plate with 200 μL in each well. Then, the cultures were cultivated at 30 °C for 36 h with fast shaking. The OD_600_ of GMS and the two mutants were quantified at a 2 h interval via an automatic growth curve analyzer (Bioscreen C, Oy Growth Curves Ab Ltd., Helsinki, Finland). This experiment was conducted with 3 independent biology duplicates.

### 2.6. Antimicrobial Activity Assays

The antimicrobial activity of GMS and its insertion mutants were examined following our previous protocols [37]. Briefly, the *Xoc* or *Xoo* solutions were added into the NA medium plates. Then the tested strain suspensions were applied onto the filter paper (5 μL). The inhibition zones were measured, and three biological replicates were performed.

### 2.7. Generation of Tol-Pal Operon Overexpression Strains

To overexpress the *tol-pal* operon in the mutant strain of Δ*csrA1A2A3*, a 4377 bp fragment containing the *tol-pal* operon and its native promoter region was amplified by PCR with primers *tol*-*pal*-F and *tol*-*pal*-R (Table S2). The PCR product was cloned into pBSPPc as an EcoRI/BamHI fragment to yield the recombinant plasmid pBS-*tol-pal.* Then it was introduced into Δ*csrA1A2A3* by electroporation and further analyzed by testing for bacteriostasis and pseudoiodinine production as described above.

### 2.8. Statistical Analysis

All the experiments were performed in triplicate to confirm reproducibility. All figures were analyzed using Graphpad Prism version 10. Data are presented as mean ± standard deviation (SD). Statistically significant differences between the two groups were analyzed by Student’s t test.

## 3. Results

### 3.1. Genome-Wide Identification of the Antibacterial Functional Genes of GMS Against Xoc RS105

**Figure 1:**
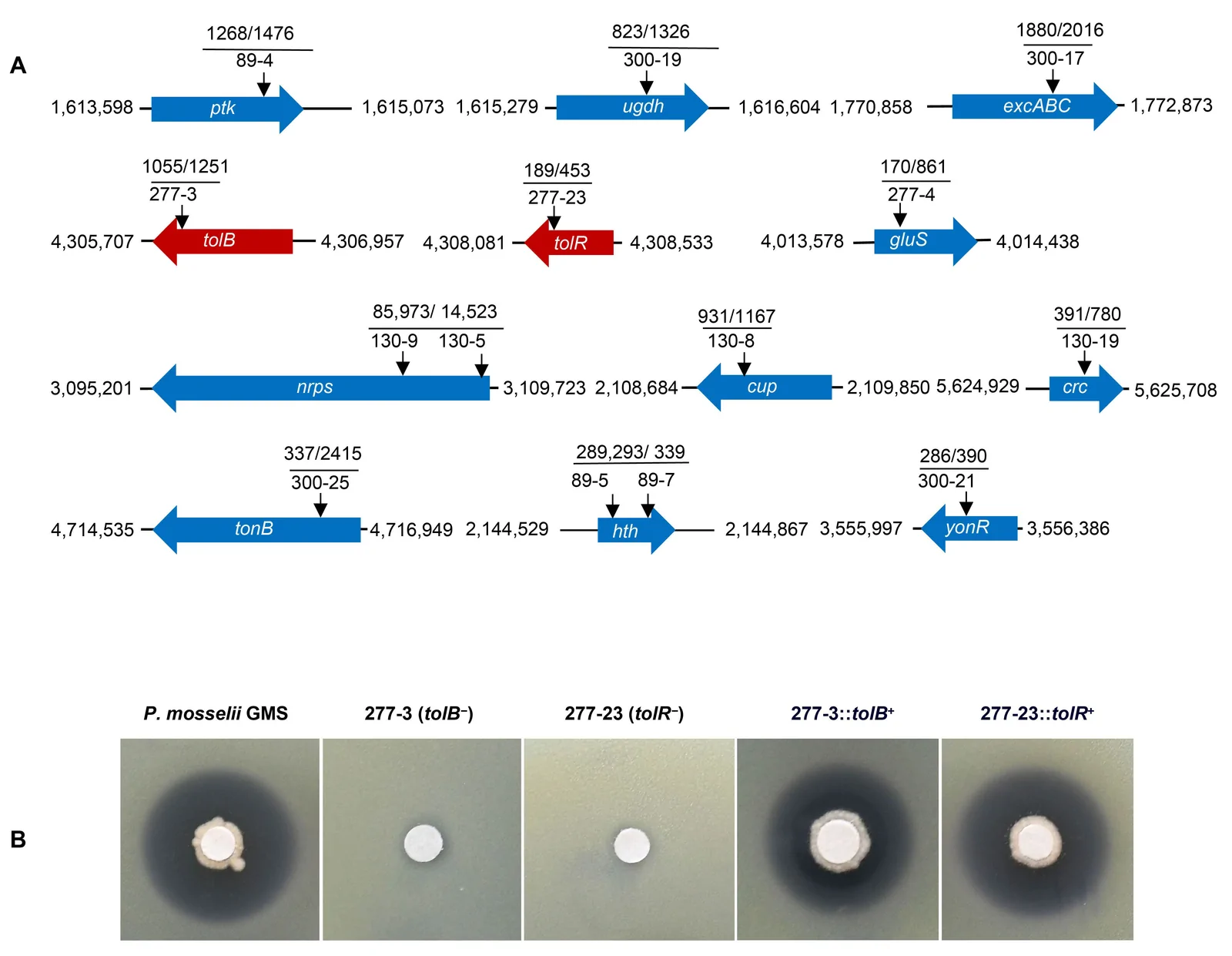
Genome-wide identification of genes involved in antibacterial activity of *Pseudomonas mosselii* GMS against *Xoc* RS105. (**A**) Characterization of Tn5 insertion mutants exhibiting altered antagonistic activity against *Xoc* RS105. Arrows indicate insertion sites of Tn5 transposon in 12 candidate genes in GMS genome. Red and blue arrows represent genes associated with complete loss or partial reduction in antagonistic activity, respectively. (**B**) Antagonistic activity of *tolB* and *tolR* mutants and their corresponding complemented strains against *Xoc* RS105. *P. mosselii* GMS as control strain.

Our previous research demonstrated that pseudoiodinine was the major antimicrobial compound produced by *P. mosselii* 923 and established a genetically modified GMS strain with an enhanced pseudoiodinine production of 22.4-fold [14]. To identify genes potentially involved in pseudoiodinine production and antibacterial activity, a Tn5-1063 transposon mutagenesis system was employed to generate random insertion mutants in the GMS strain. Approximately 15,000 random transposon insertion mutants were subsequently screened (Figure S1), and 14 mutants exhibited completely abolished or partially impaired antagonistic activity against *Xoc* RS105 (Figure S2). Sequencing analysis revealed that the Tn5-1063 insertion sites were mapped to 14 genes that located in 12 genomic regions of *P. mosselii* 923 (Figure 1A). Functional annotation analysis indicated that these genes were associated with diverse biological processes, including secondary metabolite biosynthesis (*nrps*), transcriptional regulation (*hth* and *yonR*) [38], carbohydrate metabolism (*ugdh*) [39], energy metabolism (*tonB*) [40], and cell envelope-associated functions (*tolB* and *tolR*) [41] (Table 1). Among these mutants, two independent insertions were identified in the *nrps* and *hth* genes, which resulted in reduced antagonistic activity against *Xoc* RS105. In addition, single insertions in *tyk*, *ugdh*, *excABC*, *gluS*, *cup*, *crc*, *tonB*, and *yonR* also impaired antibacterial activity. Notably, two independent insertion mutants were identified in *tolB* and *tolR*, and both mutants completely lost antagonistic activity against *Xoc* RS105. Although *nrps* and *hth* were also identified as independent insertion targets, we selected *tolB* and *tolR* for further investigation because both genes encode components of the Tol-Pal system, providing a defined genetic and mechanistic framework for investigating their potential roles in antagonistic activity and pseudoiodinine production. The potential contributions of *nrps*, *hth*, and other identified genes to antibacterial activity or pseudoiodinine production remain unclear and warrant further investigation.

To further confirm the roles of *tolB* and *tolR*, full-length copies of these genes were introduced into the corresponding insertion mutants. Complementation of *tolB* and *tolR* restored antagonistic activity against *Xoc* RS105 to levels comparable with the GMS strain (Figure 1B). Because pseudoiodinine was the only antimicrobial compound identified in *P. mosselii* 923 [14], these results indicated that *tolB* and *tolR* were essential for antibacterial activity and suggested that the Tol-Pal system may have contributed to pseudoiodinine production.

### 3.2. The Roles of tolB and tolR in Bacterial Growth

**Figure 2:**
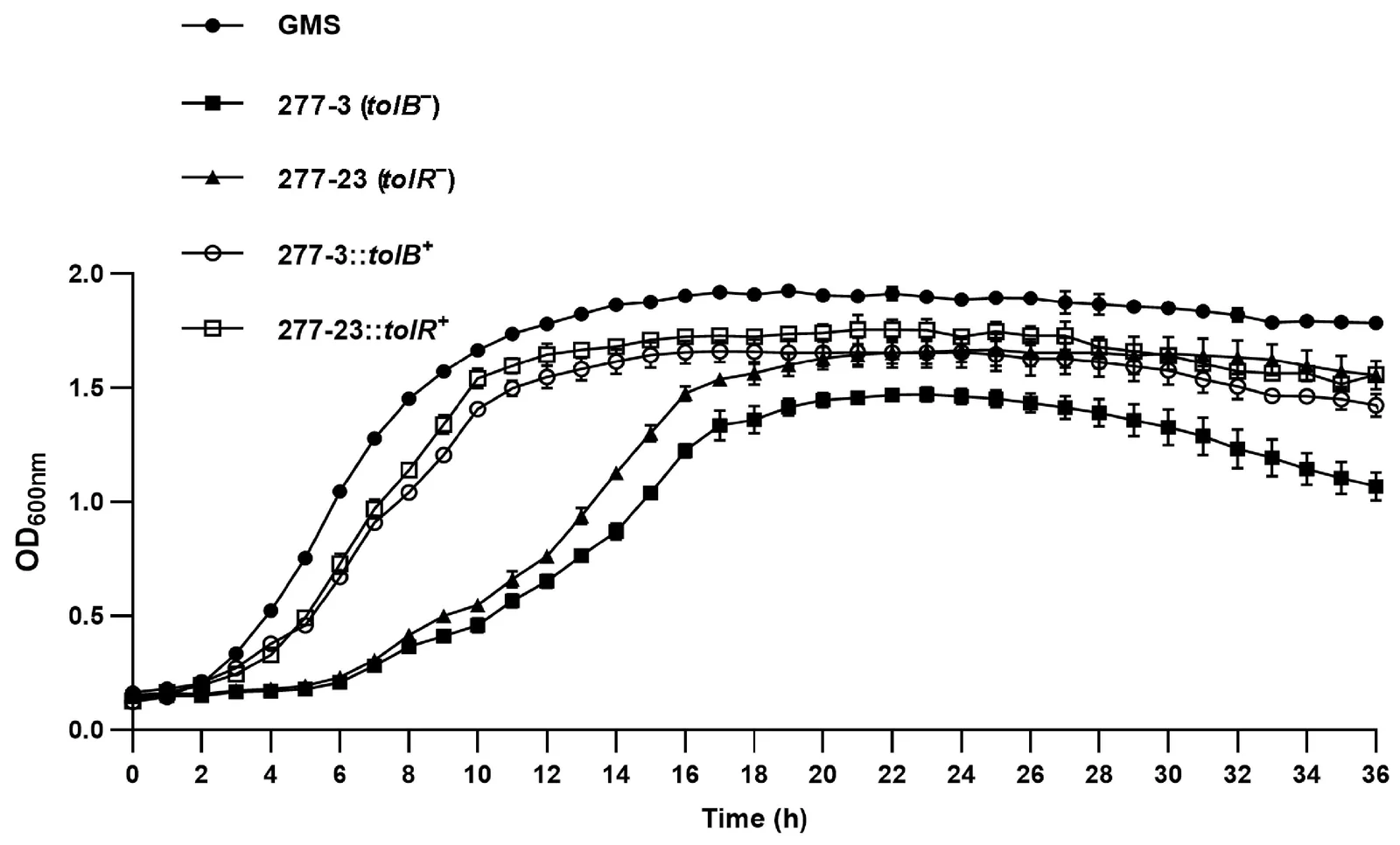
Effects of *tolB* and *tolR* mutations and complementation on the growth of *P. mosselii* GMS. Growth curves of the GMS, *tolB*, and *tolR* mutants and their corresponding complemented strains in LB medium based on OD_600_ measurements. Error bars show means ± SD (*n* = 3 biological independent replicates).

To investigate the biological roles of *tolB* and *tolR* in GMS, the growth characteristics of the corresponding insertion mutants (277-3 and 277-23) were analyzed. Compared with the GMS strain, both *tolB* and *tolR* insertion mutants exhibited significantly impaired growth during the exponential phase, whereas no significant differences in growth were observed between the two mutants (Figure 2). Importantly, the complementation of *tolB* and *tolR* substantially restored the growth of the corresponding mutants, with the complemented strains exhibiting growth kinetics comparable to those of the GMS strain during the exponential phase (Figure 2). These results demonstrated that the growth defects caused by the disruption of *tolB* and *tolR* could be largely reversed by genetic complementation, further supporting the role of the Tol-Pal system in maintaining efficient bacterial growth. Collectively, these findings suggested that the Tol-Pal system may influence antagonistic activity and pseudoiodinine production by affecting bacterial physiological status.

### 3.3. Effects of tolB and tolR Mutations on Antagonistic Activity and Pseudoiodinine Production

**Figure 3:**
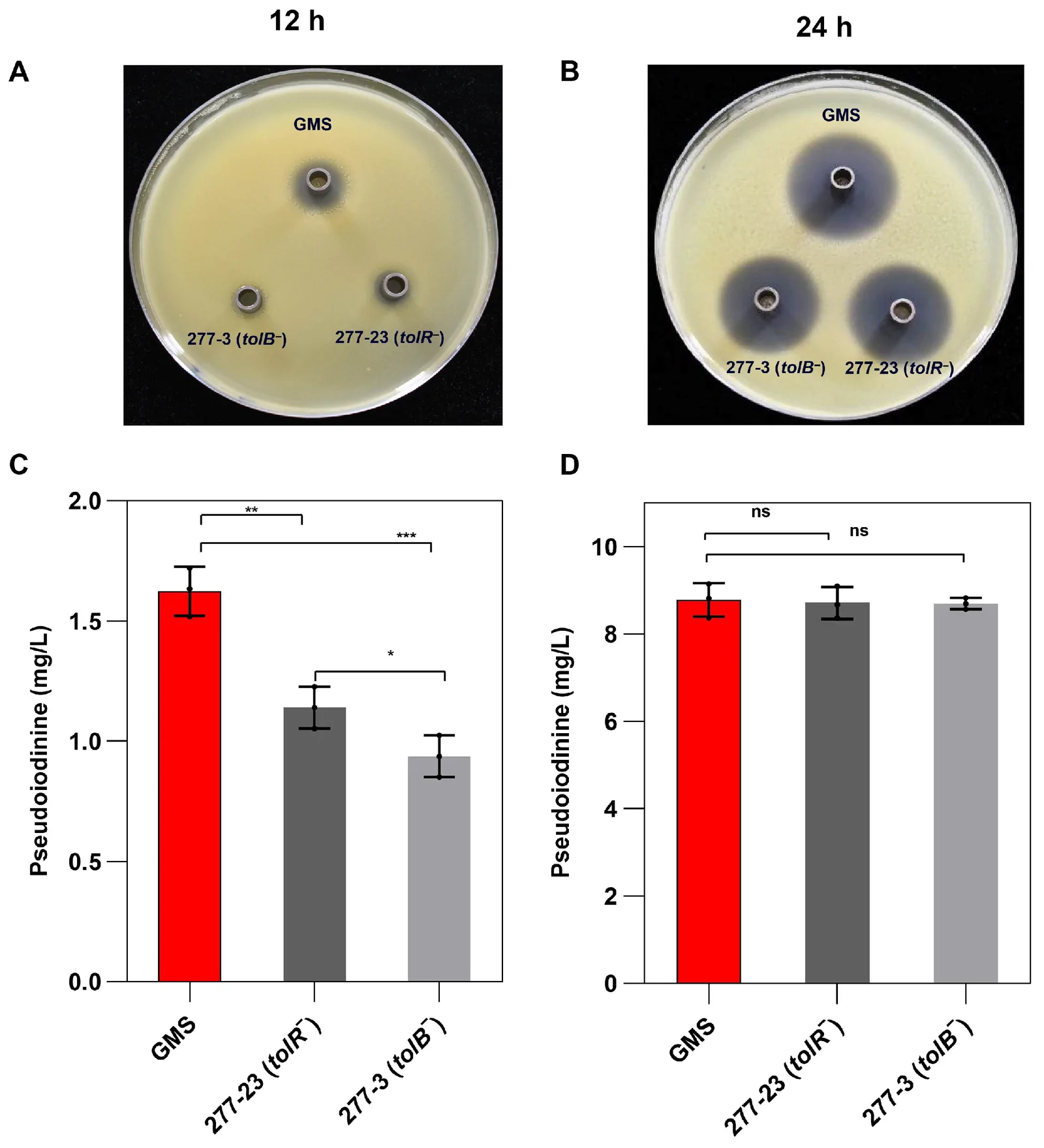
Antimicrobial activities and pseudoiodinine production assays. (**A**,**B**) Antibacterial activity assays of GMS, *tolB*, and *tolR* mutants against *Xoc* RS105 after 12 h and 24 h of fermentation. (**C**,**D**) Quantitative analysis of pseudoiodinine production of the three strains after 12 h and 24 h of fermentation, respectively. The numbers on the y-axes show pseudoiodinine yields. Error bars show means ± SD (*n* = 3 biological independent replicates); significant differences at * *p* < 0.05, ** *p* < 0.01, and *** *p* < 0.001, and ns indicates there is no significance (*p* > 0.05).

To further determine whether *tolB* and *tolR* mutations affected antagonistic activity and pseudoiodinine production, the levels of pseudoiodinine in the fermentation broth of mutants 277-3 and 277-23 were analyzed at 12 h and 24 h of fermentation, respectively (Figure 3). To exclude the potential influence of differences in bacterial biomass, the cultures were adjusted to the same OD_600_ before pseudoiodinine extraction and quantification. Thus, pseudoiodinine production was compared on an equivalent bacterial biomass basis. The antagonistic activities against *Xoc* RS105 were also evaluated to reflect the bioactivity of pseudoiodinine. At 12 h of fermentation, both *tolB* and *tolR* mutants exhibited significantly reduced antagonistic activities compared with the GMS strain (Figure 3A). However, no significant differences were observed between the GMS strain and the two mutants after 24 h of fermentation (Figure 3B). Similarly, at 12 h, both mutants exhibited reduced pseudoiodinine production compared to that of GMS strain (Figure 3C), whereas no significant differences in pseudoiodinine production were observed between the mutants and the GMS strain after 24 h of fermentation (Figure 3D). These results demonstrated that the disruption of *tolB* and *tolR* affected bacterial growth during the exponential phase, which may contribute to the reduced antagonistic activity and pseudoiodinine accumulation observed at the early fermentation stage. However, upon entering the stationary phase, no significant differences in antagonistic activity or pseudoiodinine production were observed between the mutants and the GMS strain.

Collectively, these results indicated that the Tol-Pal system was associated with growth-dependent changes in antagonistic activity and pseudoiodinine production during fermentation. Therefore, we next investigated whether genetic engineering of the *tol-pal* operon could enhance antibacterial activity and pseudoiodinine production.

### 3.4. Overexpression of the Tol-Pal Operon Enhances Pseudoiodinine Production

**Figure 4:**
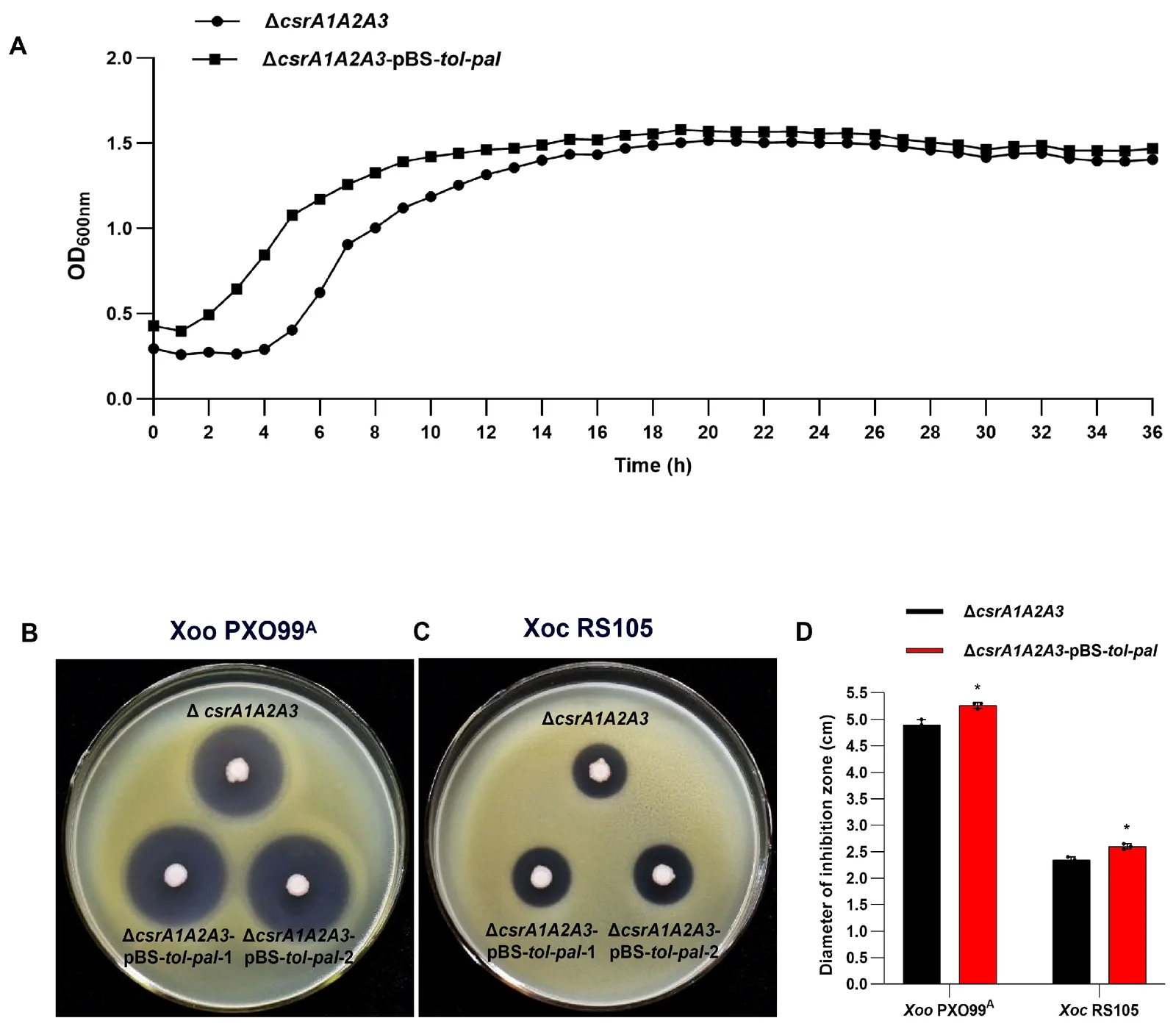
Effect of overexpression of the *tol-pal* operon. (**A**) Growth curves of Δ*csrA1A2A3* and Δ*csrA1A2A3*-pBS-*tol-pal* strains in LB medium. (**B**–**D**) Antimicrobial activity of the Δ*csrA1A2A3* and Δ*csrA1A2A3*-pBS-*tol-pal* strains against (**B**) *Xoo* PXO99^A^ and (**C**) *Xoc* RS105 and (**D**) quantitative analysis of inhibition zone diameters. The diameter of inhibition zones ± SD (cm) (*n* = 3 biologically independent plates) and significant differences at * *p* < 0.05 (one-way ANOVA followed by LSD test).

**Figure 5:**
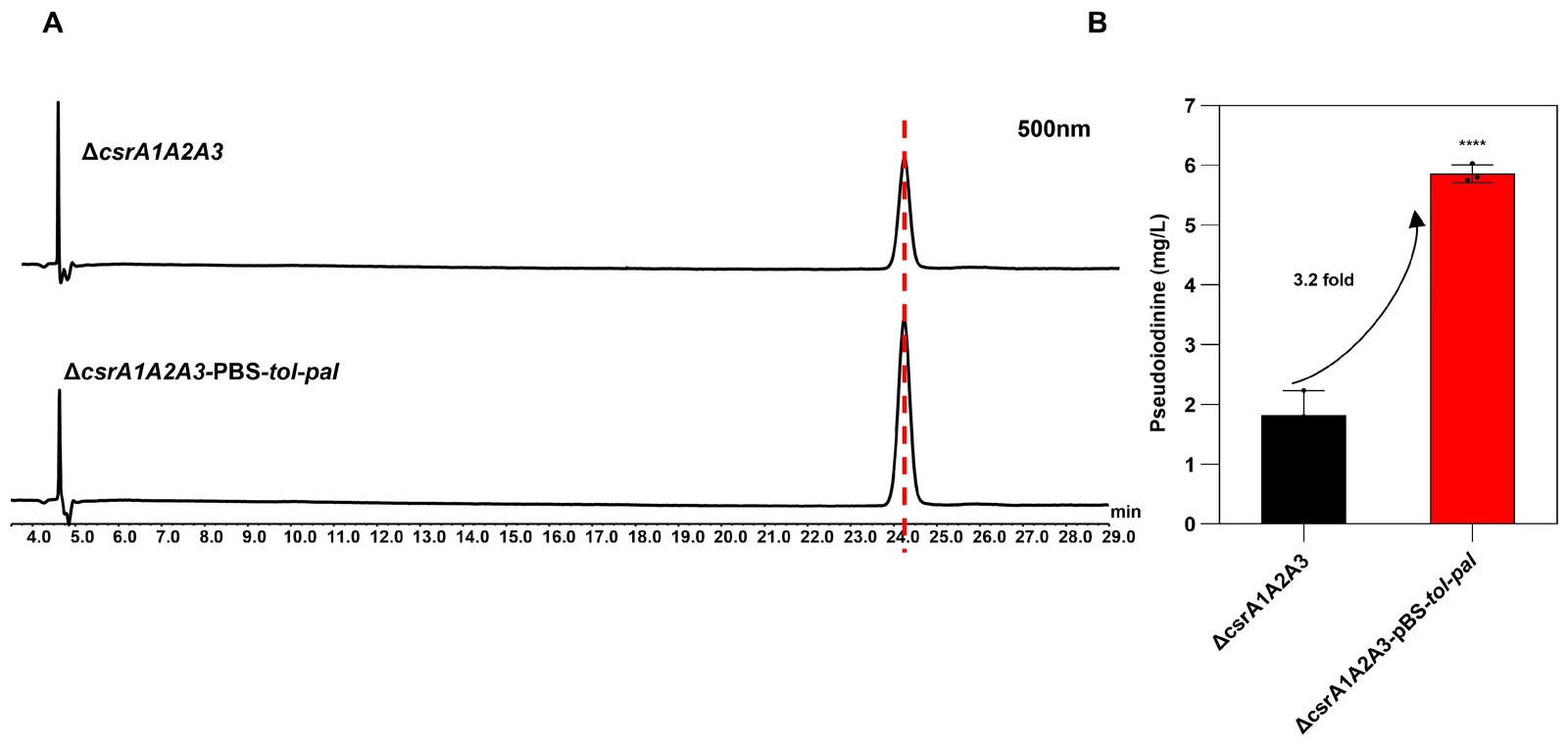
Effect of *tol-pal* operon overexpression on pseudoiodinine production. (**A**) HPLC profiles (detected at 500 nm) of the Δ*csrA1A2A3*-pBS-*tol-pal* strain compared with the Δ*csrA1A2A3* mutant. The line indicates the pseudoiodinine peak. (**B**) Quantitative analysis of the pseudoiodinine production of the two strains. The numbers on the y-axes show pseudoiodinine yields. The arrow indicates the trend of increased pseudoiodinine production. Error bars show means ± SD (*n* = 3 biological independent replicates) and significant differences are indicated by **** *p* < 0.0001.

In our previous study, we showed that the *psdABCDEFG* operon was responsible for pseudoiodinine biosynthesis and that CsrA1, CsrA2, and CsrA3 negatively regulated *psdA* expression. The deletion of *csrA1A2A3* increased pseudoiodinine production by 6.6-fold [14]. Because the GMS strain is gentamicin-resistant, the gentamicin-sensitive Δ*csrA1A2A3* mutant was selected as the parental strain for the introduction of the gentamicin-resistant pBS-*tol-pal* plasmid, thereby avoiding antibiotic resistance interference. The Tol-Pal system consists of five envelope-associated proteins, TolA, TolB, TolQ, TolR, and Pal, and is essential for maintaining outer membrane integrity and proper cell division in Gram-negative bacteria [35]. To investigate the potential function of the *tol-pal* operon in pseudoiodinine production, the *tolQRAB*-*pal* operon was introduced into the Δ*csrA1A2A3* mutant to generate the overexpression strain Δ*csrA1A2A3*-pBS-*tol-pal*. The growth curve analysis revealed that the Δ*csrA1A2A3*-pBS-*tol-pal* strain exhibited significantly enhanced growth during the exponential phase (Figure 4A). Notably, the engineered strain also exhibited significantly enhanced antibacterial activity against *Xoo* PXO99^A^ and *Xoc* RS105 compared with the parental strain (Figure 4B,D). Moreover, pseudoiodinine production in the Δ*csrA1A2A3*-pBS-*tol-pal* strain reached 5.86 mg/L, representing a 3.2-fold increase compared with the Δ*csrA1A2A3* strain (1.82 mg/L) (Figure 5A,B). These findings suggest that overexpression of the *tol-pal* gene cluster promotes pseudoiodinine production, likely through improving bacterial growth mediated by the Tol-Pal system.

## 4. Discussion

Antimicrobial secondary metabolites produced by plant-associated *Pseudomonas* species played important roles in microbial competition, ecological adaptation, and the biological control of plant diseases [9]. The biosynthesis of these metabolites were often regulated by complex regulatory networks involving quorum-sensing systems, global regulators, and environmental signals [16,17,19]. In *Pseudomonas* species, the Gac/Rsm regulatory cascade represented one of the major regulatory pathways controlling the production of diverse antimicrobial compounds, including 2,4-DAPG, phenazines, lipopeptides, and other metabolites [16]. However, the physiological mechanisms connecting bacterial envelope homeostasis and antimicrobial metabolite biosynthesis remain poorly understood. In this study, genome-wide transposon mutagenesis identified *tolB* and *tolR*, two key components of the Tol-Pal system, as important factors influencing the antagonistic activity of *P. mosselii* GMS against *Xoc* RS105 (Figure 1). Further investigations revealed that the Tol-Pal system positively regulated pseudoiodinine production by enhancing bacterial growth, uncovering a previously unrecognized connection between envelope-associated physiological regulation and antimicrobial secondary metabolism.

In addition to *tolB* and *tolR*, several other genes identified in the transposon screening showed reduced antagonistic activity against *Xoc* RS105 (Figure S2). These genes were associated with diverse biological functions, mainly including secondary metabolism (*nrps*), transcriptional regulation (*hth* and *yonR*), carbohydrate metabolism (*ugdh*), energy metabolism and membrane transport (*tonB*), and cell envelope-associated functions (*excABC*, *tolB*, and *tolR*) (Table 1). The identification of multiple functional categories suggested that the antibacterial phenotype of *P. mosselii* GMS may be influenced by both antimicrobial metabolite production and the physiological state of bacterial cells. Notably, two independent insertions were identified in both *nrps* and *hth*. NRPSs are well-established biosynthetic systems for the production of diverse antimicrobial secondary metabolites, particularly cyclic lipopeptides, in *Pseudomonas* and other bacteria [42,43]. Representative NRPS-dependent compounds include xantholysin, entolysin, putisolvin, and white line-inducing principle (WLIP), which have been associated with antagonistic activities [44]. Thus, disruption of the identified *nrps* gene may reduce the production of antimicrobial metabolites and consequently contribute to decreased antagonistic activity against *Xoc* RS105. However, whether the *nrps* gene is directly involved in pseudoiodinine biosynthesis or affects antibacterial activity through other antimicrobial metabolites remains unclear. Similarly, the identified *hth* gene is predicted to encode a helix-turn-helix (HTH)-type transcriptional regulator. HTH-containing regulators can control the expression of genes involved in diverse cellular processes. For example, ExsA, a master transcriptional regulator of the type III secretion system (T3SS) in *P. aeruginosa*, contains an HTH DNA-binding motif that mediates DNA recognition and the transcriptional activation of T3SS-associated genes [45]. Therefore, disruption of *hth* may alter the expression of downstream genes involved in metabolism or other cellular processes, thereby affecting antibacterial activity. However, the specific regulatory targets of *hth* and its potential relationship with pseudoiodinine production have not been determined in the present study. Thus, the potential contributions of *nrps*, *hth*, and other identified genes to pseudoiodinine production warrant further investigation. Future studies integrating pseudoiodinine quantification, metabolomic profiling, and the genetic characterization of these mutants will help determine whether their effects on antagonistic activity are mediated by pseudoiodinine or other antimicrobial compounds [46].

In contrast, *tolB* and *tolR* were selected for detailed investigation because both genes encode components of the Tol-Pal system, and their independent disruption resulted in a complete loss of antagonistic activity, which was restored by genetic complementation. Together with the observed growth defects and their reversal upon complementation, these findings provided stronger genetic and physiological evidence supporting a role of the Tol-Pal system in maintaining bacterial growth and promoting pseudoiodinine production.

The Tol-Pal system is a highly conserved envelope-associated multiprotein complex in Gram-negative bacteria and has been extensively characterized for its essential roles in maintaining outer membrane integrity, bacterial normal growth [20], coordinating cell division, and promoting bacterial survival under environmental stresses [47]. Nevertheless, whether the Tol-Pal system contributed to the regulation of antimicrobial secondary metabolite production remained largely unknown. In this study, disruption of *tolB* and *tolR* significantly impaired the growth of *P. mosselii* GMS, particularly during the exponential phase. Importantly, genetic complementation substantially restored the growth of both mutants toward the level of the GMS strain (Figure 2), suggesting that an intact Tol-Pal system was important for efficient bacterial growth. Similar phenotypes have been observed in *S. Choleraesuis* [31] and *E. chrysanthemi* [34]. Loss of Tol-Pal function may therefore increase envelope stress and disrupt energy allocation, ultimately restricting bacterial growth and physiological adaptation [31]. These findings emphasized that bacterial growth fitness and envelope homeostasis were important physiological determinants influencing the expression of beneficial microbial traits.

Secondary metabolite production in bacteria was closely associated with cellular physiological status, and alterations in growth conditions, nutrient availability, and metabolic activity can significantly influence the accumulation of bioactive compounds [48]. In many *Pseudomonas* species, antimicrobial metabolites were typically produced during the transition from active growth to the stationary phase and were regulated by complex interactions between global regulatory networks and cellular metabolic states [9,49]. In *P. mosselii*, pseudoiodinine biosynthesis has been reported to be controlled by regulatory pathways involving the Gac/Rsm system [14]. However, our results indicated that the Tol-Pal system affected pseudoiodinine production through a distinct mechanism. Interestingly, although disruption of *tolB* and *tolR* severely impaired bacterial growth during the exponential phase (Figure 2), no significant differences in pseudoiodinine production were detected during the stationary phase (Figure 3). These results indicated that Tol-Pal was unlikely to directly regulate the pseudoiodinine biosynthetic pathway but may influence metabolite production by maintaining optimal cellular fitness and growth capacity. Similar indirect effects have been observed in various microorganisms, where disruptions of fundamental cellular processes, including membrane integrity and energy metabolism, altered secondary metabolite production by affecting precursor availability and metabolic flux rather than directly controlling biosynthetic gene expression [50,51]. Therefore, our findings suggest that the Tol-Pal system may primarily promote pseudoiodinine biosynthesis by maintaining optimal physiological conditions rather than acting as a direct biosynthetic regulator, complementing previously characterized transcriptional regulatory mechanisms.

To further investigate the contribution of the Tol-Pal system to pseudoiodinine production, we constructed a strain overexpressing the *tol-pal* operon and observed a significant increase in antagonistic activity and pseudoiodinine production compared with the parental strain (Figure 4 and Figure 5). This finding demonstrated that improving bacterial physiological fitness represented an effective strategy for enhancing secondary metabolite accumulation. Unlike conventional metabolic engineering approaches that mainly target biosynthetic genes or precursor pathways [52,53], our findings highlighted the potential of targeting global physiological processes to enhance antimicrobial metabolite biosynthesis. However, the production level of pseudoiodinine in the engineered strain remained relatively limited, indicating that additional optimization was required before practical agricultural or industrial applications. Future studies should focus on integrating metabolic engineering strategies, fermentation optimization [54], co-cultivation approaches [55], and advanced genome-editing tools such as the CRISPR/Cas systems [56] to further improve production efficiency. Moreover, elucidating the molecular connections among the Tol-Pal system, central metabolism, and pseudoiodinine biosynthetic regulation will provide deeper insights into how envelope-associated physiological processes influence secondary metabolism. Evaluating the ecological fitness and biocontrol performance of engineered strains under field conditions will also be essential for assessing their practical application potential. Collectively, this study reveals a previously unrecognized link between bacterial envelope homeostasis and antimicrobial metabolite production, highlighting the Tol-Pal system as a promising physiological engineering target for developing *Pseudomonas* strains with enhanced biocontrol potential.

## 5. Conclusions

In conclusion, this study identified the Tol-Pal system as an important physiological determinant contributing to pseudoiodinine production in *P. mosselii*. Genome-wide transposon mutagenesis and functional analyses demonstrated that mutations in *tolB* and *tolR* impaired bacterial growth during the exponential phase and reduced antagonistic activity at the early fermentation stage, whereas these effects were alleviated during the stationary phase. Furthermore, overexpression of the *tol-pal* operon significantly enhanced pseudoiodinine production, indicating that the Tol-Pal system promotes antimicrobial metabolite accumulation primarily by maintaining bacterial growth and physiological fitness. Collectively, these findings reveal a previously unrecognized connection between envelope-associated physiological regulation and antimicrobial secondary metabolism in *P. mosselii*, providing new insights into the optimization of beneficial microorganisms for sustainable agricultural applications.

## Figures and Tables

**Table 1:** Genes involved in *P. mosselii* 923 antibacterial activity against *Xoc* RS105.

Gene ID	Gene Name	COG Function	KEGG Pathway
gene1426	*ptk*	Protein tyrosine kinase	Not assigned
gene1940	*hth*	Helix-turn-helix domain-containing protein	Not assigned
gene2877	*nrps*	Non-ribosomal peptide synthetase	Metabolism of terpenoids and polyketides
gene1901	*cup*	Cupin 4 family protein 50S ribosomal protein L16 3-hydroxylase	Translation
gene5089	*crc*	Catabolite repression control protein Crc	Replication and repair
gene3909	*tolB*	Tol-Pal system beta propeller repeat protein TolB	Membrane transport
gene3911	*tolR*	Biopolymer transport protein TolR	Membrane transport
gene 3637	*gluS*	Glutathione S-transferase Glutathionyl-hydroquinone reductase	Not assigned
gene1587	*excABC*	Excinuclease ABC subunit B	Replication and repair
gene1427	*ugdh*	UDP-glucose/GDP-mannose dehydrogenase family protein	Carbohydrate metabolism
gene3237	*yonR*	SPBc2 prophage-derived uncharacterized HTH-type transcriptional regulator YonR	Not assigned
gene4305	*tonB*	TonB-dependent siderophore receptor	Membrane transport

## Data Availability

The original contributions presented in this study are included in the article. Further inquiries can be directed to the corresponding author.

## Supplementary Materials

The following supporting information can be downloaded at: https://www.mdpi.com/article/10.3390/microorganisms14092079/s1, Figure S1. Representative antagonistic activity of transposon insertion mutants against Xoc RS105. Figure S2: Antagonistic activity of 14 mutants against Xoc RS105. Table S1: Strains and plasmids used in this study. Table S2: Primers used in this study.

## Abbreviations

The following abbreviations are used in this manuscript:

GMS	Genetically modified strain
*Xoc*	*Xanthomonas oryzae* pv. *oryzicola*
*Xoo*	*Xanthomonas oryzae* pv. *oryzae*
