# Supplementary material for: The Tol-Pal System Promotes Pseudoiodinine Production by Enhancing Bacterial Growth in Pseudomonas mosselii
Source: Microorganisms. 2026 Sep 17;14(9):2079. doi: 10.3390/microorganisms14092079 (PMC13609540; doi:10.3390/microorganisms14092079)

Supplementary Material

1. Supplementary Tables

Table S1 | Strains and plasmids used in this study.

Strains and plasmids	Relevant characteristics	Source
**Strains**		
RS105	The wild type of *Xanthomonas oryzae* pv. *oryzicola*	This study
PXO99^{A}	The wild type of *X. oryzae* pv. *oryzae*	This study
GMS	The genetically-modified strain of Δ*csrA1A2A3*-pBSPPc-P2064-RBS-Pseu-ORF	This study
Δ*csrA1A2A3*	The *csrA1A2A3* triple deletion mutant of *Pseudomonas mosselii* 923	This study
277-3	*tolB* Tn5 insertion mutant of GMS	This study
277-23	*tolR* Tn5 insertion mutant of GMS	This study
89-4	*tyk* Tn5 insertion mutant of GMS	This study
89-5/7	*hth* Tn5 insertion mutant of GMS	This study
130-5/9	*nrps* Tn5 insertion mutant of GMS	This study
130-8	*cup* Tn5 insertion mutant of GMS	This study
130-19	*crc* Tn5 insertion mutant of GMS	This study
277-4	*gluS* Tn5 insertion mutant of GMS	This study
300-17	*excABC* Tn5 insertion mutant of GMS	This study
300-19	*udgh* Tn5 insertion mutant of GMS	This study
300-21	*yonR* Tn5 insertion mutant of GMS	This study
300-25	*tonB* Tn5 insertion mutant of GMS	This study
DH5α	F-, endA1, thi-1, recA1, Φ80lacZ, Δ M15	This study
**Plasmids**		
pRL1063a	Containing Tn5 transposon, Km^r	Gang wang's lab[a]
pBSPPc	$oriT^+$ replacement vector derived from pBR322, Gm^r, $Carb^r$	Shuangjun Lin's lab[b]
pBS-*tolB*	*tolB* with its native promoter cloned in pBSPPc, Gm^r, $Carb^r$	This study
pBS-*tolR*	*tolR* with its native promoter cloned in pBSPPc, Gm^r, $Carb^r$	This study
pBS-*tol-pal*	Tol-pal synthesis gene cluster contained gene *tolQ* promoter cloned in pBSPPc by EcoRI and BamHI, Gm^r, $Carb^r$	This study

a University of Henan, Henan, China; b University of Shanghai Jiao Tong, Shanghai, China;

Note: Km^r, kanamycin resistance, Gm^r, gentamycin resistance, $Carb^r$, carbenicillin

Table S2 | Primers used in this study.

Primers	Sequence 5' to 3'	Description
M13-F	CGCCAGGGTTTTCCCAGTCAC	Primers for confirmation of the pBSPPc-based construct
Gm-R	CCGCAGTGGCTCTCTATAC	
*tolB*1-com-F	TTCCTGCAGCCCGGGGGATCCCCTGTTGTTCGTGGTCCATTC	Amplifies a 282-bp fragment of *tolQ* promoter
*tolB*1-com-R	GCCATGGCCATGCTTATTCTCCTGCGTTCGATT	
*tolB*2-com-F	AGAATAAGCATGGCCATGGCAGAAGAAAA	Amplifies a 1281-bp fragment of *tolB* gene
*tolB*2-com-R	CACCTAAGCGGCCGCTCTAGATCAGTTCAGGTAAGGGGACCAG	
*tolR*1-com-F	TTCCTGCAGCCCGGGGGATCCCCTGTTGTTCGTGGTCCATTC	Amplifies a 282-bp fragment of *tolQ* promoter
*tolR*1-com-R	ACTCGGGCCATGCTTATTCTCCTGCGTTCGATT	
*tolR*2-com-F	AGAATAAGCATGGCCCGAGTTCGCCAC	Amplifies a 453-bp fragment of *tolR* gene
*tolR*2-com-R	CACCTAAGCGGCCGCTCTAGATCAGGGCGCCTCGGTAAT	
tol-pal-F	CGCGGATCC CCTGTTGTTCGTGGTCCATTC	Amplifies 4377-bp of *tol-pal* operon and its promoter
tol-pal-R	CCGGAATTC TTACTTACGCAGTTCTACGCGACG	
Tn5-1063-F	AGGAGGTCACATGGAATATCAGAT	Specific primers used for Tn5 transposon sequencing
Tn5-1063-R	TACTAGATTCAATGCTATCAATGAG	
Tn5-F	TTTGTTCGGCTTGGTATCGC	Primers for confirmation of the Tn5 transposon
Tn5-R	ATTGCTTAGGTCCATTCTCA	

Note: Underlined bases indicate restriction enzyme sit

2. Supplementary Figures

Figure S1. Representative antagonistic activity of transposon insertion mutants against *Xoc* RS105. A subset of transposon insertion mutants (~15,000 mutants screened in total) were selected.

Antagonistic activity was determined by measuring inhibition zones against *Xoc* RS105.

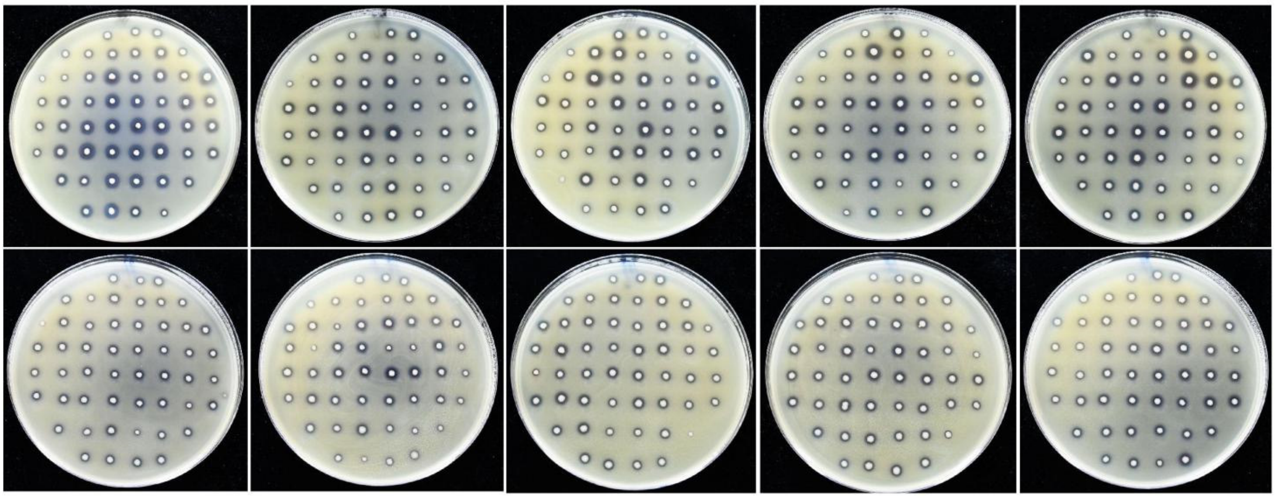

Figure S2. Antagonistic activity of 14 mutants against *Xoc* RS105. *P. mosselii* GMS as the control strain.

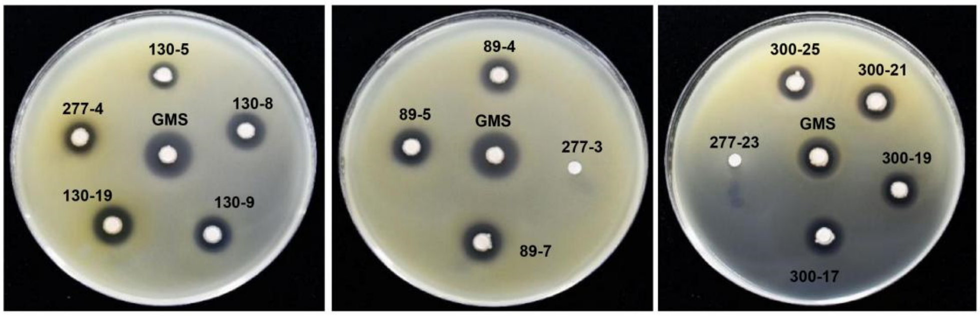

Supplement: Supplementary file 1 [file microorganisms-14-02079-s001.zip › microorganisms-4568481-supplementary.pdf]
